# Assessment of Liver Function With MRI: Where Do We Stand?

**DOI:** 10.3389/fmed.2022.839919

**Published:** 2022-04-06

**Authors:** Carolina Río Bártulos, Karin Senk, Mona Schumacher, Jan Plath, Nico Kaiser, Ragnar Bade, Jan Woetzel, Philipp Wiggermann

**Affiliations:** ^1^Institut für Röntgendiagnostik und Nuklearmedizin, Städtisches Klinikum Braunschweig gGmbH, Braunschweig, Germany; ^2^Institut für Röntgendiagnostik, Universtitätsklinikum Regensburg, Regensburg, Germany; ^3^MeVis Medical Solutions AG, Bremen, Germany

**Keywords:** liver function, MRI, T1 relaxometry, deep learning, artificial intelligence

## Abstract

Liver disease and hepatocellular carcinoma (HCC) have become a global health burden. For this reason, the determination of liver function plays a central role in the monitoring of patients with chronic liver disease or HCC. Furthermore, assessment of liver function is important, e.g., before surgery to prevent liver failure after hepatectomy or to monitor the course of treatment. Liver function and disease severity are usually assessed clinically based on clinical symptoms, biopsy, and blood parameters. These are rather static tests that reflect the current state of the liver without considering changes in liver function. With the development of liver-specific contrast agents for MRI, noninvasive dynamic determination of liver function based on signal intensity or using T1 relaxometry has become possible. The advantage of this imaging modality is that it provides additional information about the vascular structure, anatomy, and heterogeneous distribution of liver function. In this review, we summarized and discussed the results published in recent years on this technique. Indeed, recent data show that the T1 reduction rate seems to be the most appropriate value for determining liver function by MRI. Furthermore, attention has been paid to the development of automated tools for image analysis in order to uncover the steps necessary to obtain a complete process flow from image segmentation to image registration to image analysis. In conclusion, the published data show that liver function values obtained from contrast-enhanced MRI images correlate significantly with the global liver function parameters, making it possible to obtain both functional and anatomic information with a single modality.

## Introduction

The liver is responsible for several functions in the body, including the primary detoxification of various metabolites, synthesis of proteins, and production of digestive enzymes (1); it also has a central role in carbohydrate and lipid metabolism. The primary functions of the liver include the production and excretion of bile and the detoxification and purification of the blood. Therefore, hepatic diseases need to be taken seriously. Liver diseases encompass a variety of diseases, especially metabolic dysfunction associated with fatty liver disease, alcohol-associated liver disease, viral hepatitis, and autoimmune liver diseases (2). These pathologies can cause fibrosis and may progress to cirrhosis, resulting in chronic liver disease (CLD). Cirrhosis is a major risk factor for the development of hepatocellular carcinoma (HCC) (3). Both are classified as global health burdens and together account for 3.5% of all deaths worldwide (4–6). In the clinic, a routine liver function of patients with CLD needs to be regularly controlled. Furthermore, for patients undergoing major liver resection, the function of the hepatic remnant needs to be assessed preoperatively to avoid posthepatectomy liver failure (PHLF). The latest advances in liver surgery and perioperative care have considerably improved patient outcomes following hepatectomy (7). Nevertheless, with morbidity rates of 10–40%, PHLF remains a major factor producing a poor prognosis (8). Therefore, precise preoperative assessment of liver function plays a crucial role in clinical decision-making. The need to measure liver function has led to the development of innovative methods to assess liver function. An outline of the newest methods is reviewed in this article. With a focus on the most promising MRI strategies, particular attention is given to the use of automation and artificial intelligence (AI) in liver imaging.

## Current Scoring Systems and Global Liver Function Tests

Liver biopsy is currently considered the gold standard for the evaluation of liver diseases. However, it has drawbacks, including invasiveness, costliness, and low patient acceptance. Furthermore, it is subject to sampling errors and examiner experience (9). In clinical practice, liver function and the severity of liver disease are evaluated based on clinical signs and biochemical blood parameters, such as bilirubin, alkaline phosphatase, glutamyl transferase, aspartate transaminase, alanine transaminase, albumin, and prothrombin time (1, 10). These are rather static tests reporting the current state of the liver without regarding changes in liver function (10). Nonetheless, in day-to-day routine testing, they are suitable for assessing the severity of liver damage and are thus an integral part of various scoring systems.

Comprehensive scoring systems for evaluating the liver function, such as the Child-Pugh (CP) score and the model for end-stage liver disease (MELD) score, have been developed. The CP Grading System is used for uniformly describing and classifying liver cirrhosis into different stages according to symptom severity. The ranking into the three CP groups (A-C) is based on a point scale (11); the CP score is calculated based on three objective [serum albumin, serum bilirubin, and international normalized ratio (INR)] and two subjective (ascites and encephalopathy) parameters. The subjective parameters vary with the use of diuretics or paracenteses in the treatment of ascites and the treatment of encephalopathy with lactulose (12). Therefore, in recent years, it has become common practice to use the MELD score to describe the severity of liver diseases (13), as no subjective parameters are considered. The MELD score is especially used in the allocation of organs for liver transplantation; it helps identify and prioritize the care of patients in acutely life-threatening situations due to liver disease and/or whose treatment is of utmost urgency. The MELD score is calculated using the following objective parameters: serum bilirubin, serum creatinine, and INR, from 6 to 40 points; the higher the score is, the lower the patient's probability of surviving the next 3 months without a liver transplant (14). The CP score and MELD score assess global liver function and are useful in determining whether patients with HCC and cirrhosis are candidates for resection or transplantation, but they are unable to determine the safe extent or removal (15). While they can roughly estimate the risks of performing a hepatectomy, they are not appropriate as a diagnostic tool in the preoperative environment.

To some extent, this also applies to the indocyanine green (ICG) test and the ^13^C-methacetin breath test (^13^C-MBT), dynamic tests that nevertheless are clinically useful in assessing global liver function. ICG clearance is currently the most widely used quantitative liver function test (16). ICG is a tricarbocyanine dye that binds to plasma proteins (albumin and α1-lipoprotein) and becomes evenly distributed in the blood within 2–3 min after intravenous injection. It is excreted into the bile exclusively via the liver without intrahepatic conjugation (17); its elimination is dependent on liver blood flow, hepatic cell function, and excretion via the biliary system. After administration, the blood ICG level decreases exponentially for ~20 min, at which time ~97% of the dye is excreted. ICG clearance is determined by serum sampling or pulse dye densitometry with an optical sensor on the finger (18, 19); commonly related parameters include the ICG retention ratio after 15 min (ICG-R15) and the plasma disappearance rate (ICG-PDR) (19). A large retrospective study showed that ICG clearance is associated with postoperative liver dysfunction; a PDR value <19.5% and an R15 value >5.6% were identified as cutoff values for identifying patients who are more likely to have a worse outcome for both minor and major hepatic resections (20). The ICG-R15 value corresponds to liver blood flow and hepatic functional reserve; in cirrhosis, it is used as a prognostic marker in decompensated cirrhotic patients and candidates for liver resection surgery (21). In hepatic surgery, such as liver resection and liver transplantation, the ICG elimination test is used as a liver function test to evaluate patient outcomes, as a prognostic marker, and as a diagnostic tool (22). However, the ICG test has limitations that hinder its use as a universal liver function test (23–26).

The ^13^C-MBT, like the ICG test, is a dynamic liver function test that reflects the patient's actual liver function at the time of the measurement. The ^13^C-MBT is based on the activity of the cytochrome P450 1A2 (CYP1A2) enzyme system, expressed exclusively and distributed evenly in the liver (27). The agent ^13^C-methacetin is metabolized exclusively by the CYP1A2 system, which converts it to paracetamol and ^13^CO_2_. The exhaled ^13^CO_2_ produces a change in the normal ^13^CO_2_/^12^CO_2_ ratio in the exhaled air and can be analyzed with an infrared spectroscopic detector. Therefore, the ^13^C-MBT provides quantitative information about liver function. The liver maximum capacity (LiMax) value (28) and a decision tree algorithm for hepatectomy that was developed from it (29) can be used to preoperatively evaluate a patient for liver surgery and better estimate the postoperative outcome. A preoperative LiMax value below 80 μg/kg/h for the future liver remnant increases the risk of PHLF (29). However, this is feasible only when the LiMax value is combined with, for example, CT volumetry to determine the volume of the liver and the future liver remnant. However, this test also has some limitations that need to be considered in its application (30). Regardless, a LiMax value above 315 μg/kg/h is assumed to indicate normal liver function, whereas a value below 140 μg/kg/h indicates a severe impairment of liver function (29). However, although the ^13^C-MBT, like the previously mentioned ICG test, provides functional data for the entire liver, it does not provide data for the functional activity of specific liver regions. The inhomogeneous distribution of liver function can be described by scintigraphic methods (31). For patients, a decisive disadvantage of scintigraphic imaging procedures is the constant risk of radiation exposure. In addition, several other liver function values can be found in the literature that has also found their way into clinical practice, such as the Makuuchi algorithm (decision algorithm for the surgical treatment of HCC) or values derived from FibroScan (ultrasound elastography) (32–35) (for an overview of the values, refer to Table 1). However, imaging techniques remain superior for visualizing the liver and provide additional important information about vascular anatomy. For instance, MRI techniques do not expose the patient to radiation and allow good visualization of organs and soft tissues. The development of liver-specific contrast agents such as gadoxetic acid has led to the development of several approaches for determining liver function that has been published in recent years. Moreover, studies have already shown that contrast-enhanced MRI is superior to CT (36) in the detection of intrahepatic recurrent HCC after surgery (37) and to dynamic CT in the detection and diagnosis of HCC (38).

**Table 1:** Overview of liver-related scores and tests.

	**Rational**	**Literature**
Child Pugh (CP) score	Bilirubin total [mg/dL], serum albumin [g/dL], INR and evaluation of ascites and encephalopathy	Pugh et al. (11), Child et al. (102)
MELD	Bilirubin total [mg/dL], serum creatinine [mg/dL], INR	Kamath et al. (13), Malinchoc et al. (103)
Indocyanine green (ICG) test	Fluorescence dye; parameters: ICG-R15 [%], ICG-PDR [%/min], ICG clearance [ml/min/m^2^] and ICG half-life time [min]	Sakka (19), Hunton et al. (104)
^13^C-methacetin breath test (^13^C-MBT)	^13^CO_2_/^12^CO_2_ ratio after metabolization of ^13^C-methacetin	Stockmann et al. (28, 29)
Makuuchi algorithm	Historical: ICG-15, serum bilirubin, presence of ascites; actually, based on CP score, number and size of tumors.	Kokudo et al. (32), Makuuchi et al. (33)
ALBI grade	Bilirubin [μmol/L], Albumin [g/L]	Johnson et al. (105)
BILCHE score	Bilirubin total [mg/dL], serum cholinesterase [U/L]	Donadon et al. (106)
FibroScan	Liver stiffness measurement (LSM) [kPa]; controlled attenuation parameter (CAP) [dB/m]; FibroScan-AST (FAST)	Newsome et al. (34), Sandrin et al. (35)
MRI – Gd-EOB-DTPA	Signal intensity measurement or T1 relaxometry from MR images before and after contrast agent administration.	see Table 2

*INR, international normalized ratio; AST, aspartate aminotransferase*.

## Contrast Agents in Liver MRI

**Figure 1:**
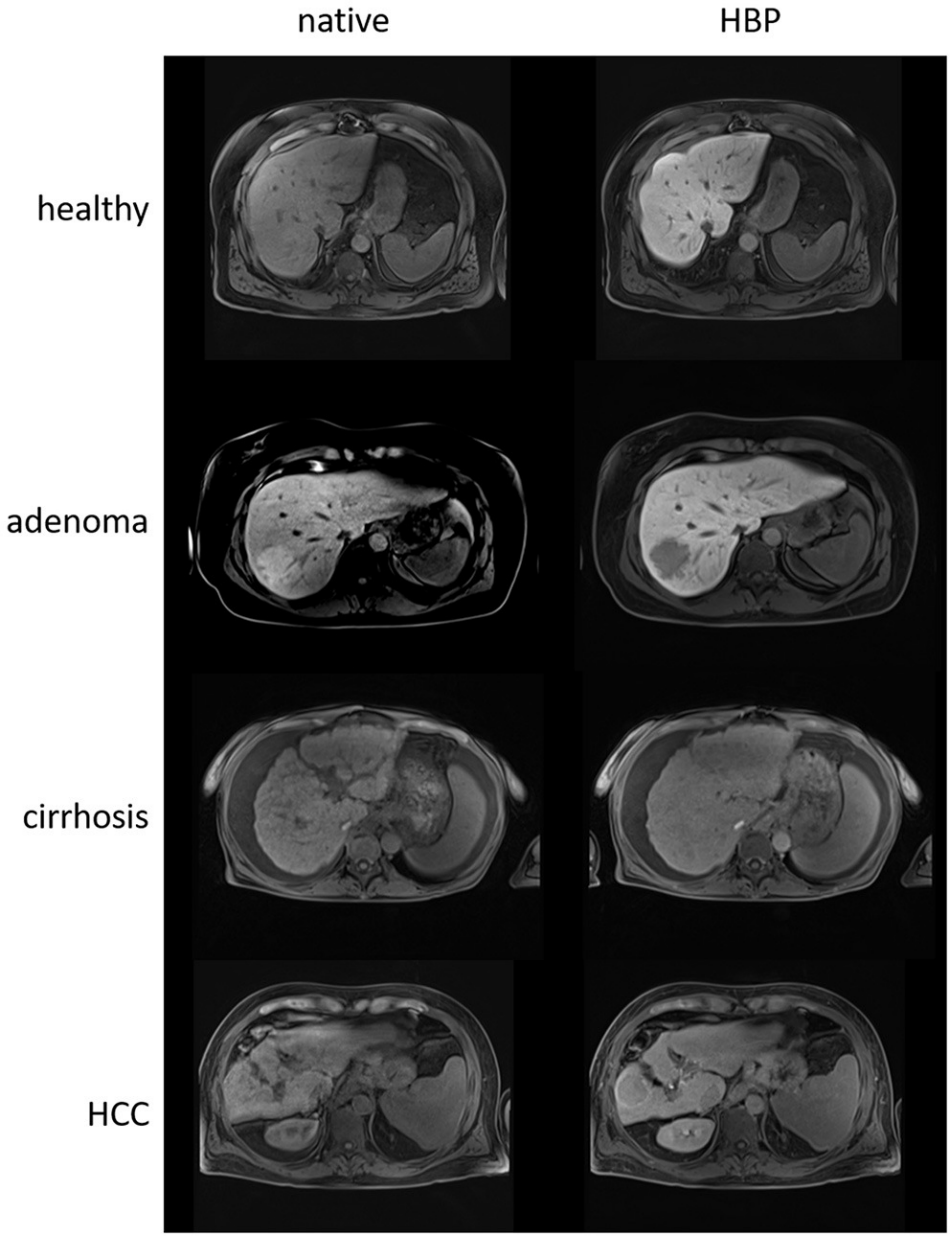
Native and hepatobiliary-phase (HBP) T1 images of a healthy liver (56 years, male), a liver with an adenoma in liver segment VII (48 years, female), a cirrhotic liver (60 years, female), and a cirrhotic liver with a hepatocellular carcinoma (HCC) in liver segment VI (59 years, male) using gadoxetic acid as the contrast agent. The healthy liver clearly appears more hyperintense than the cirrhotic liver in the HBP image relative to the native image due to the ability of more functional hepatocytes to take up the contrast agent. Both adenoma and HCC appear hypointense in the HBP images.

Magnetic resonance imaging is capable of generating variable image contrast using different pulse sequences. In this process, the image parameters corresponding to the longitudinal (T1) and transverse (T2) relaxation times and the signal intensities on T1- and T2-weighted images vary depending on certain tissue properties (39). The intensity of these signals can be enhanced by contrast agents, such as gadolinium-based contrast agents, which have been established for use in liver MRI. Gadolinium (Gd) is a highly paramagnetic element that reduces the T1, T2, and T2^*^ relaxation times of surrounding water protons (40). Here, T1 shortening plays a very useful role in enhancing the signal intensity. In the clinical use for liver examination, there are two classes of contrast agents, namely, nonspecific extracellular and specific intracellular (hepatobiliary) agents. Nonspecific extracellular agents are taken up by the hepatic artery or portal vein, distribute rapidly in the extracellular space, and are almost exclusively excreted by glomerular filtration. They have no protein-binding properties and are used to assess the perfusion, blood flow, and vascularity of the liver (41). In contrast, hepatobiliary agents are taken up by functioning hepatocytes and excreted through the bile (41). This characteristic allows visualization of nonfunctioning hepatocyte lesions such as liver adenomas and HCC, which appear hypointense on hepatobiliary-phase images (Figure 1) and thus allow a functional assessment of the liver. Only two hepatobiliary agents, namely, gadoxetic acid (Gd-EOB-DTPA) and gadobenic acid (Gd-BOPTA), are in clinical use. However, Gd-BOPTA is less frequently used due to, among other reasons, its elimination half-life of 1–2 h and the fact that only 3–5% is cleared through biliary excretion. Gadoxetic acid enters hepatocytes via members of the organic anion transporting polypeptide (OATP) protein family; specifically, OATP1 B1 and B3 are responsible for transport into the liver (42). Biliary excretion from hepatocytes is performed by the multidrug resistance-associated protein 2 (MRP2) transporter. Approximately 50% of the administered Gd-EOB-DTPA is excreted via the kidney, and the rest is cleared via the OATP/MRP route (41). As a result of the greater hepatic uptake, liver parenchymal enhancement reaches its maximum (hepatobiliary phase, HBP) after 15–20 min, while for Gd-BOPTA, it starts after 1 h (40), making Gd-EOB-DTPA the preferred contrast agent. Consequently, both uptake and excretion of gadoxetic acid allow the quantification of regional liver function. However, it should be mentioned that both uptake and excretion can be influenced by the altered expression of OATPs and MRPs, either due to genetic factors or liver disease (43–47). Nonetheless, in early studies with Gd-EOB-DTPA, it became clear that enhancement could be useful for detecting liver lesions in HBP images (48). In 2010, Tajima et al. first suggested that the degree of enhancement may reflect liver cellular function (49).

## Recent Approaches to Liver Function Measurement Using Contrast-Enhanced MRI

Contrast-enhanced MRI enables both the characterization of liver lesions and the assessment of regional liver function. In addition to functional information, it provides important anatomical information, e.g., for surgical planning, including lesion volume and vascular supply. In other words, contrast-enhanced MRI could be used as a one-stop examination to assess liver function. Recent relevant literature reveals a variety of equations and names for liver function measurements based on contrast-enhanced MRI data, which can be somewhat confusing at first glance. Essentially, however, two parameters are measured for assessing liver function, namely, signal intensities or the T1 relaxation time.

### Signal Intensity-Based Indices

To measure signal intensities (SIs), up to two MRI sequences are needed, one acquired before intravenous contrast agent application and one obtained during the HBP, both of which are normally part of the standard MRI liver protocols. The SI data are obtained using manually drawn regions of interest (ROIs) on the corresponding MR image. Typically, several ROIs are drawn for the liver, and the mean value is used for the calculations. The easiest and most convenient way to calculate the SI is simply the quotient of the SI before and after the administration of the contrast agent or to use only the HBP image and correlate the SI of the liver to a reference value (Table 2) (50). Here, the appropriate references are the SIs of the spleen, muscles, or portal vein. This correction is necessary because the SI has a nonlinear relationship with the contrast agent concentration (51). Several studies have shown that the liver-to-spleen, liver-to-muscle, and liver-to-portal vein ratios correlate with liver function markers such as biochemical blood parameters, prothrombin activity, CP score, or MELD score (52–55). Nevertheless, the most widely applied SI measurement method is a relative enhancement (RE), whose calculation does not require a reference value. The ROIs are drawn over a variety of liver segments to calculate the mean SI, ideally avoiding the heterogeneity caused by image artifacts or inhomogeneous disease distribution. The SI value of the unenhanced image is subtracted from the SI of the HBP image and then divided by the SI of the unenhanced image (Table 2). In a retrospective study with 110 patients, Haimerl et al. showed that both the RE and the SI of the HBP are highly correlated with the result of the ^13^C-MBT and can thus be used to determine the liver function (56). Likewise, Elkilany et al. demonstrated that RE can be used to assess global and regional liver function, showing that it was highly correlated with blood parameters and the MELD score. Moreover, the authors found that RE might be used to monitor disease progression in patients with sclerosing cholangitis (57). In the literature, a normalized version of the RE has also been described, where the values from liver-to-reference are used for the calculation (58). In addition, a further calculation method is used that takes liver volume into account (hepatocellular uptake index, HUI, Table 2); this, in turn, is better correlated with global liver function, measured with the ICG-PDR, than other SI-based indices (59, 60). This is particularly useful for determining the function of the liver remnant before hepatectomy, which, as noted earlier, is necessary to avoid PHLF. The remnant HUI (rHUI) can be used as a predictor of PHLF, as Tsujita et al. showed in a retrospective study of 41 patients who underwent hepatectomy (61). With a slightly different approach, Asenbaum et al. showed that the function of the future liver remnant may be a good predictor for PHLF in a study involving 62 patients. For the calculation, they used the RE and weight-adapted volumes of the liver remnants (62). In both studies, liver volume was measured using CT scans. However, a volumetric determination can also be performed with MRI data, as shown in the study by Ippolito et al. (55). Kudo et al. opted for a more sophisticated approach using a 3D volumetric analysis system (63). By using a semiautomatic approach and an image processing algorithm, they extracted the liver and spleen parenchyma by placing volumes of interest; the resulting extracted 3D volumes allowed measurement of the average SI and determination of the liver-to-spleen ratio. In the study, the authors enrolled 181 patients and revealed a strong correlation of the liver-to-spleen ratio with CP score, ICG-R15, blood parameters, and histological findings, among others.

**Table 2:** Summary of equations for assessing liver function.

**SI quotient (107)**	** $\frac{SI_{post}}{SI_{pre}}$ **
**Liver to reference (spleen, muscle or portal vein) (52–55)**	** $\frac{SI_{post\;liver}}{SI_{post\;reference}}$ **
**Relative enhancement (RE) (48, 108, 109)**	** $\frac{SI_{post}-SI_{pre}}{SI_{pre}}$ **
**Normalized RE (58)**	** $\frac{\frac{SI_{post\;liver}}{SI_{post\;reference}}-\frac{SI_{pre\;liver}}{SI_{pre\;reference}}}{\frac{SI_{pre\;liver}}{SI_{pre\;reference}}}$ **
**Hepatocellular uptake index (HUI) (60)**	** $liver\;volume\;\times \left[\left(\frac{SI_{post\;liver}}{SI_{post\;spleen}}\right)-1\right]$ **
**Reduction rate of T1 relaxation time (rrT1) (65, 66)**	** $\left[\left(\frac{{T1}_{pre}-{T1}_{post}}{{T1}_{pre}}\right)\right]\times 100\;\left(\%\right)$ **

*SI, signal intensity; post, hepatobiliary phase/enhanced after contrast agent administration; pre, unenhanced; reference, spleen, muscle, or portal vein*.

However, a question that has not yet been answered is which of these indices is superior to the others. In a retrospective study with 287 patients, Beer et al. showed that the SI-based indices correlated with each other and that none was superior (RE, HUI, SI quotient, and liver-to-spleen). They also showed that these indices had good inter- and intrareader agreement (64). However, this is still a controversial issue, as the SI measurements are relative values and depend on technical parameters such as the receiver coil, the gain of the radio frequency amplifier, and the pulse sequence designed by the different vendors (65–69); additionally, as mentioned earlier, there is no linear relationship between the gadolinium concentration and the MR SI (51, 59). Nevertheless, newer studies have shown that apparently neither scanner model nor scanner field strength (1.5 T or 3T) affects the reproducibility of the data (52, 70). In contrast, values measured by T1 relaxometry are not affected at all by these different factors and yield absolute, comparable values (59). In addition, studies comparing several SI-based indices and T1 relaxation values have shown that the reduction rate of the T1 relaxation correlates better with ICG-PDR (59) or ICG-R15 (71) than the SI-based indices.

### T1 Relaxometry

In this context, the term relaxometry refers to the measurement of relaxation times. In particular, the T1 relaxation time is relevant for the evaluation of liver function. The T1 relaxation time is a measurement of the speed at which the nuclear spin magnetization returns to its equilibrium state after a radiofrequency pulse. Thus, the T1 relaxation time depends on the energy transfer rate of the excited protons toward the surrounding environment. Unlike SI, which is measured in an arbitrary unit, the T1 relaxation time, whose unit is milliseconds, is a quantifiable unit and, in theory, is directly related to the concentration of contrast agent in hepatocytes (52, 72). To generate T1 maps, different methods can be used; however, the best-proven ones are the variable flip angle (VFA) with B1 inhomogeneity correction and the look-locker inversion recovery (LLIR) sequence.

Kim et al. showed that the two methods are equivalent, although the VFA technique yields higher T1 values than the LLIR method, which is why caution is advised, as the resulting liver function values may not be interchangeable (73). As previously mentioned, the T1 relaxation time reduction rate (rrT1, Table 2) has been found to be better correlated with global liver function parameters than other T1 relaxation time or SI indices, as shown by comparative studies (59, 71, 74). For this reason, in particular, the rrT1 has gained importance as a liver function parameter. To determine the T1 values, analogous to the SI measurements, ROIs are drawn manually on the corresponding images, and the mean value is used for calculation. In a retrospective study involving 65 patients, the rrT1 was shown to decrease with the severity of liver fibrosis as assessed by the Metavir score, a metric based on biopsy results. The authors demonstrated that the rrT1 value correlated significantly with the fibrosis stage, which could be differentiated with a sensitivity of 78% and a specificity of 94%, (75). Other studies using, e.g., the ^13^C-MBT have also demonstrated that the rrT1 continuously decreases with increasing progression of liver failure (76, 77). Although the correlation with ^13^C-methacitin is highly significant, the authors of reference (76) also found a slightly lower correlation than that of the rrT1 value with ICG-PDR from their previous work (65). This is thought to be due to the different metabolic pathways of the substrates. Whereas ICG and gadoxetic acid are similarly eliminated via the OATP/MRP route, ^13^C-methacetin, in contrast, is metabolized via the CYP1A2 system (27, 42, 78). Nevertheless, the rrT1 value can serve as an indicator of liver disease progression. Moreover, in an early study with 233 patients, a cutoff value of 50% for rrT1 was proposed for differentiating patients with normal liver function (MELD score ≤ 10) from those with impaired liver function (MELD score ≥ 11) (79). To date, this cutoff score has not been validated, not least because the attention in previous studies has focused on the methodology and the establishment of a liver function value itself. Overall, the literature suggests that a value below 50% is indicative of poor liver function.

In addition to the aforementioned findings, it is also feasible to measure the rrT1 value per liver segment, as shown in the study by Zhou et al.. In their study of 103 patients classified by CP score, they showed that the segmental rrT1 values differed within each group (CP-C from 40.6 to 55.5%; CP-B from 47.9 to 70.7%) (80). Additionally, according to the studies mentioned earlier, a decrease in rrT1 was observed with increasing disease severity. Regrettably, that study did not consider the different sizes of different liver segments. However, several studies have demonstrated that liver volume-adjusted rrT1 values correlated better with ICG-PDR (65) and ICG-R15 (67) than the rrT1 values alone. In addition, Yoon et al. indicated that the combined value of T1 and liver volume, adjusted for patient weight, could serve to identify patients with CP-A cirrhosis at high risk of decompensation (81). Moreover, by comparing the T1 values of the left and right liver lobes, they showed a heterogeneous distribution of liver function (median, right lobe: 407 ms and left lobe: 372 ms) (81). Elsewhere, different T1 relaxation values for different liver segments have also been shown (71).

In patients scheduled for hepatectomy, to avoid PHLF, it is necessary to assess the precise liver function of the remnant liver, especially in those with liver diseases (82). By combining rrT1 with the remnant liver volume, good diagnostic accuracy was obtained (67). Additionally, Huang et al. showed the importance of the rrT1 value of the liver remnant, where the rrT1 value of the remnant was an independent risk factor for major postoperative complications (83); specifically, in patients who have undergone a major partial hepatectomy, the lower the rrT1 value, the higher the risk of a postoperative complication was. However, the authors indicated that neither the ICG-R15 value nor the remnant liver volume alone could serve as a postoperative complication risk factor. They also demonstrated that HBP and T1 mapping images can be used for virtual hepatectomy to determine the volume and T1 relaxation time of the remnant using a computer-assisted semiautomatic approach but noted that this required a long processing time. Consequently, Bastati et al. proposed a visual scoring system, the functional liver imaging score (FLIS) (84), derived from contrast-enhanced MRI. They were able to show that the FLIS could identify patients at increased risk of a first hepatic decompensation and mortality (85), but the dependence on the rater has not yet been investigated. The extent to which this score will be used in the clinic remains to be determined.

These examples demonstrate the importance of the combination of functional and morphological parameters in therapy selection and outcome. T1 relaxometry is still not used in standard workup protocols in clinical practice because T1 mapping software is still in the investigation phase. Additionally, T1 mapping with B1 correction is part of the licenses of some MRI vendors (e.g., Siemens Healthineers using MapIT license) and has been used in a number of the presented studies (71, 73, 77). Nevertheless, the rrT1 must still be calculated manually; thus, there is a demand for software solutions to establish the rrT1 value as a liver function parameter, ideally in prospective trials. In addition, 3D volumetry for the liver is not yet fully automated; however, various software solutions for 3D volumetry are available on the market, one of which was successfully used by Kudo et al. (63) to semiautomatically determine the liver-to-spleen ratio. As in this case, semiautomatic or manual approaches are mostly used for volume determination. Automatic segmentation, e.g., based on AI approaches, could improve the consistency of the results. Furthermore, AI approaches can potentially reduce the physician's workload and support the diagnostic process.

## Automated Image Analysis and AI Approaches For Liver Function Quantification

**Figure 2:**
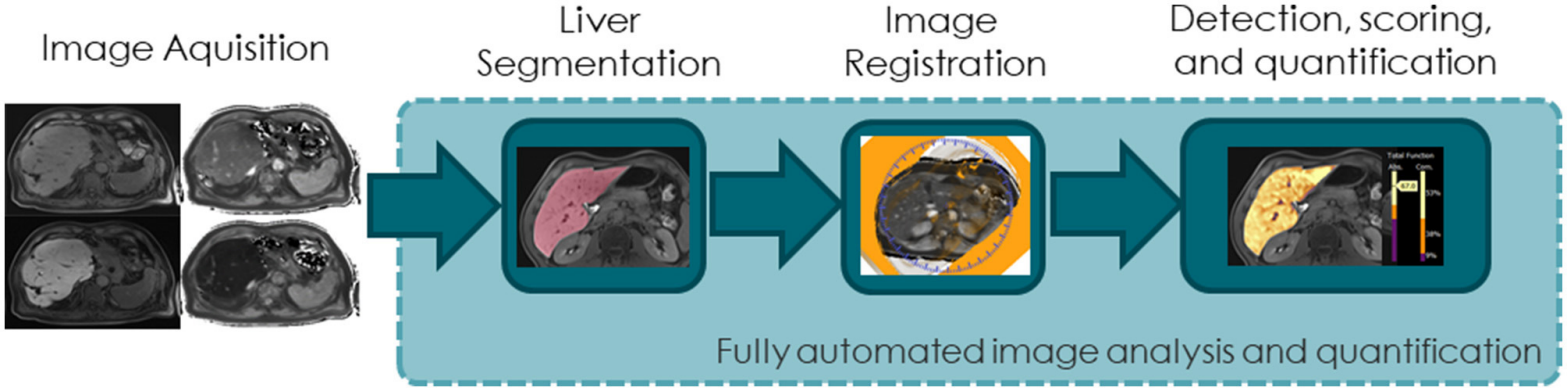
Diagram illustrating the steps needed for automated image analysis to quantify liver function.

Several steps that need to be developed in terms of automating image analysis to quantify liver function are as follows: (A) liver segmentation and volumetry; (B) image registration of native and contrast-enhanced scans; (C) detection, scoring, and quantification; and (D) fully automated MRI image-based liver function quantification (Figure 2). Both conventional image processing methods and novel AI approaches are suitable for this purpose. AI applications are increasing in popularity in medical research; further insight into AI in medical imaging is provided in the Excursus Box.

Box 1 **Excursus Box**

The term AI is used to indicate intelligence applied machine wise, in contrast to human intelligence, which is natural. Machine learning (ML) is a subfield within AI that refers to computer algorithms that automatically improve themselves through experience and the use of data. The resulting models are trained using training data and can then process additional data to make predictions. In the field of medical imaging, artificial neural networks, especially convolutional neural networks (CNNs) based on deep learning algorithms, are widely used as ML models (97). The review by Lundervold et al. provides an overview of the technological advances, including deeper insight into the network architectures, and the associated challenges in deep learning approaches focusing on MRI (98). Additionally, a good overview of CNNs in liver medical imaging is provided by the review by Zhou et al. However, they mainly address papers describing algorithms that implement CT or ultrasound imaging (99). nnU-Net was developed as a tool to address the segmentation challenge; it has strong generalization properties, does not require expert knowledge or computational resources beyond standard network training, and is currently considered the state of the art (100). In addition to image segmentation, the second key issue in image analysis for determining liver function is the alignment of images in the spatial domain to ensure proper correlation of signal intensities or relaxation times between native and contrast-enhanced scans. Classic image registration algorithms and CNN architectures have been developed and applied to various images and tasks (101).

When searching PubMed with the terms MRI, liver, and CNN, approximately 50 articles published in the last few years were retrieved, highlighting the novelty of the field. These studies reveal that the dominant topics are segmentation and classification for diagnostic support. For instance, both liver segmentation and volume determination can be performed automatically, with an intraclass correlation coefficient of 0.987 (86). Likewise, acceptable Dice coefficients comparing AI with manual segmentation between 0.91 and 0.95 have been observed (87–89). Even approaches using the same liver segmentation model for different imaging modalities, such as CT and MRI, have been successful (90). Other studies have focused more on the detection or classification of liver lesions or disease, which is important for the development of computer-aided detection or diagnosis (CADe and CADx) systems. By using CNN models, one study showed that it was possible to distinguish fibrosis stages F2 to F4 with high diagnostic performance (AUC: F4, 0.84; F3, 0.84; and F2, 0.85) (91). CNN models can also detect liver cirrhosis at the expert level, indicating the feasibility of assisting the radiologist in diagnosis (92). Beyond that, however, the majority of studies focus on the differentiation of liver lesions (87, 93, 94) or detection (94, 95) or grading of HCC (96). These proof-of-concept studies describe good-performing models with AUCs over 0.90 in some cases and thus the potential to assist radiologists in diagnosis. Although none of these studies address the determination of liver function, they demonstrate the value of AI approaches in liver diagnostics using radiological methods. Consequently, it is assumed that future research and publications will address the automation of MRI image-based liver function quantification.

## Commentary

One point that has not yet been addressed is the duration and cost-effectiveness of MRI procedures. The sequences needed for T1 relaxometry lengthen the patient's examination by <1 min. However, it may take time even for an experienced radiologist to manually determine SI or T1 relaxometry. Given the AI-based IT solutions outlined earlier, it can be assumed that this will one day be an automatic process. In addition to personnel costs, there are also licensing costs to carry out T1 relaxometry. The T1 relaxometry sequences can be easily incorporated into the existing liver MR exam as a part of the patient care, eliminating the need for an additional exam. However, although the aforementioned examples of AI in liver diagnosis and imaging illustrate the power of these approaches, they have not yet found their way into clinical practice, in part due to the lack of clinical validation. In addition, open-source data are desirable to allow objective evaluation and comparison of different methods and approaches, which is not yet possible. Thus, the answer to our initial question, “where do we stand in determing liver function?,” is as follows: liver function determination by MRI is possible, and rrT1 seems to be the best candidate. Although the presented studies demonstrate that values (SI, RE, and rrT1) obtained from contrast-enhanced MRI are significantly correlated with global liver function parameters, there is no cutoff value for stratifying patients thus far. Therefore, large prospective studies are needed to establish them as functional parameters, as most works to date have been based on retrospective studies. Of course, it would be desirable to implement software capable of segmentation and determining diagnostic values such as rrT1. However, given the rapid development of AI software in recent years, this is likely to be a minor problem moving forward.

## Author Contributions

CR and PW conceived this manuscript. All authors contributed to the content and co-authored the manuscript. Each author agrees to be responsible for the content work. All authors contributed to the article and approved the submitted version.

## Funding

The authors are part of a collaborative project sponsored by the German Federal Ministry of Education and Research as part of the program “Image-based diagnostics” (Grant number: 13GW0363A-C).

## Conflict of Interest

MS, JP, NK, RB, and JW were employed by MeVis Medical Solutions AG. The remaining authors declare that the research was conducted in the absence of any commercial or financial relationships that could be construed as a potential conflict of interest.

## Publisher's Note

All claims expressed in this article are solely those of the authors and do not necessarily represent those of their affiliated organizations, or those of the publisher, the editors and the reviewers. Any product that may be evaluated in this article, or claim that may be made by its manufacturer, is not guaranteed or endorsed by the publisher.
